# MicroRNA-149 Inhibits Proliferation and Cell Cycle Progression through the Targeting of *ZBTB2* in Human Gastric Cancer

**DOI:** 10.1371/journal.pone.0041693

**Published:** 2012-10-29

**Authors:** Ying Wang, Xiushan Zheng, Zhiyong Zhang, Jinfeng Zhou, Guohong Zhao, Jianjun Yang, Limin Xia, Rui Wang, Xiqiang Cai, Hao Hu, Cailin Zhu, Yongzhan Nie, Kaichun Wu, Dexin Zhang, Daiming Fan

**Affiliations:** 1 State Key Laboratory of Cancer Biology and Xijing Hospital of Digestive Disease, Fourth Military Medical University, Xi'an, Shaanxi, China; 2 Department of Thoracic Surgery, General Hospital of PLA Chengdu Military District, Chengdu, Sichuan Province, China; Vanderbilt University Medical Center, United States of America

## Abstract

An increasing body of evidence indicates that miR-149 can both suppress and promote tumor growth depending on the tumor type. However, the role of miR-149 in the progression of gastric cancer (GC) remains unknown. Here we report that miR-149 is a tumor suppressor in human gastric cancer. miR-149 expression is decreased in GC cell lines and clinical specimens in comparison to normal gastric epithelial cell and tissues, respectively. The expression levels of miR-149 also correlate with the differentiation degree of GC cells and tissues. Moreover, ectopic expression of miR-149 in gastric cancer cells inhibits proliferation and cell cycle progression by down-regulating *ZBTB2*, a potent repressor of the ARF-HDM2-p53-p21 pathway, with a potential binding site for miR-149 in its mRNA's 3′UTR. It is also found that ZBTB2 expression increases in GC cells and tissues compared to normal gastric epithelial cell and tissues, respectively. Silencing of *ZBTB2* leads to suppression of cell growth and cell cycle arrest in G0/G1 phase, indicating that ZBTB2 may act as an oncogene in GC. Furthermore, transfection of miR-149 mimics into gastric cancer cells induces down-regulation of ZBTB2 and HDM2, and up-regulation of ARF, p53, and p21 compared to the controls. In summary, our data suggest that miR-149 functions as a tumor suppressor in human gastric cancer by, at least partially through, targeting *ZBTB2*.

## Introduction

Gastric cancer (GC) is one of the most common cancers and major causes of cancer death worldwide, especially in Eastern Asia, according to the latest global estimation published in 2011 [1]. Despite a remarkable decrease in GC incidence in most parts of the world, there is still a great burden from the large number of GC cases and deaths worldwide. The carcinogenesis of GC is a very complicated process [2]–[5] involving the dysregulation of multiple genes [6]–[8], including oncogenes [9]–[14] and tumor suppressors [15]–[18]. However, the molecular mechanisms required for GC development and progression need to be further explored.

MicroRNAs (miRNAs) are a group of endogenously expressed, non-coding small RNAs (20–25 nucleotides in length) known to negatively regulate gene expression by suppressing translation or decreasing the stability of mRNAs by directly binding to the 3′-untranslated region (3′-UTR) of target mRNAs [19], [20]. Accumulating evidence indicates that miRNAs play important roles in development, metabolism, proliferation, differentiation, and apoptosis. In addition, aberrant post-transcriptional regulation of mRNAs by miRNAs is related to tumorigenesis [21]. Abnormal expression profiles of miRNAs have been detected in various types of human tumors, including lung [22], breast [23], prostate [24], liver [25], colon [26], and gastric cancer [27]. Moreover, some miRNAs can function as oncogenes or tumor suppressors by regulating the expression of target genes which play important roles in cell cycle progression, apoptosis, or proliferation. miR-22 [28], miR-101 [29], and miR-7 [30] have all been shown to be downregulated in tumor specimens and function as tumor suppressors; miR-17 [31] and miR-21 [32] have been shown to be upregulated in tumor specimens and function as oncogenes. These studies indicate that dysregulation of miRNAs is frequently involved in carcinogenesis and cancer progression.

Recent studies have suggested that miR-149 may play an important role in various diseases, including the progression of malignant tumors. miR-149 has been shown to function as both a tumor suppressor [33] and an oncogene [34] in the development of multiple types of solid tumors. For example, loss of miR-149 leads to gain of function of some oncogenes and correlate with tumor grade in renal cell carcinoma [35], astrocytomas [36], and prostate carcinoma [37]. Ectopic expression of miR-149 induces apoptosis of neuroblastoma and HeLa cells [33]. Elevated expression of miR-149 has been reported to be important in the progression of nasopharyngeal carcinoma [38] and melanoma metastasis [34]. Furthermore, dysregulation of miR-149 is also implicated in some non-neoplastic diseases. For example, downregulation of miR-149 expression is involved in the development of primary myelofibrosis, polycythemia vera, and essential thrombocythemia [39], and the loss of miR-149 can suppress hepatitis C virus (HCV) RNA abundance [40]. However, the expression pattern and role of miR-149 in the development of GC remains unclear.

In the present study, we found that miR-149 is significantly downregulated in GC cell lines and clinical samples in comparison to the normal gastric epithelial cell and adjacent non-tumor tissues, respectively. Ectopic expression of miR-149 inhibits proliferation and induces G0/G1 arrest of AGS and SGC7901 cells *in vitro* by targeting *ZBTB2*. Furthermore, depletion of *ZBTB2* by siRNA results in inhibition of proliferation and cell cycle arrest. The expression pattern of miR-149 and ZBTB2 in GC cell lines and clinical samples were inversely correlated, further suggesting that *ZBTB2* is a target gene of miR-149. Introduction of miR-149 results in alterations in the expression of p53, p21, ARF, and HDM2, members of the ARF-HDM2-p53-p21 pathway which is regulated by ZBTB2. In summary, these results confirm that miR-149 is downregulated in GC cells and clinical samples and suggest that miR-149 functions as a suppressor of gastric cancer cell growth by inhibiting proliferation and cell cycle progression.

## Results

### Expression of miR-149 is downregulated in GC cell lines and clinical samples

**Figure 1:**
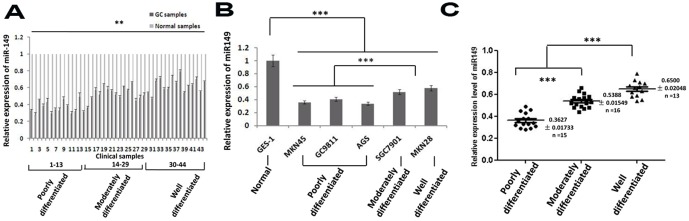
miR-149 expression is downregulated in gastric cancer cell lines and clinical samples. A. expression of miR149 in 5 human gastric cancer cell lines relative to the normal human gastric epithelial cell line,GES-1, was measured by quantitative RT-PCR (The values indicate the mean ±SEM, n = 3,t-test,*, p<0.05;*** p<0.001). B. expression of miR149 in 44 human gastric cancer clinical samples relative to their paired normal samples was measured by quantitative RT-PCR (The values indicate the mean ±SEM, n = 3, t-test, ** p<0.01). C. comparison of relative expression of miR149 in poorly, moderately and highly differentiated human gastric cancer clinical samples. (The values indicate the mean ±SEM, n = 3, One-way ANOVA analysis, F = 65.391, *** p<0.001).

To assess the role of miR-149 in the carcinogenesis of GC, we first used quantitative RT-PCR to measure the expression of miR-149 in human GC cell lines (MKN45, GC9811, AGS, SGC7901, and MKN28) and found that miR-149 was downregulated in GC cell lines compared to a normal gastric epithelial cell line, GES-1 (Fig. 1A). In particular, the expression level of miR-149 was positively correlated with the differentiation degree of GC cells. Namely, the expression level of miR-149 in poorly differentiated cell lines, such as MKN45, GC9811, and AGS, is significantly lower than in moderately and well-differentiated cell lines. The expression of miR-149 in moderately differentiated cells, SGC7901, is lower than that in the well-differentiated cell line, MKN28 (Fig. 1A).

In order to determine if levels of miR-149 also correlate with the differentiation degree of tumors we examined the expression of miR-149 in 44 human GC clinical specimens, including 13 poorly, 16 moderately, and 15 well differentiated samples. We found that the expression of miR-149 in gastric tumors is remarkably lower than in matched normal adjacent tissues (Fig. 1B). Moreover, the expression of miR-149 in more differentiated tumors was higher than in less differentiated tumors (Fig. 1C, F = 65.391, *p*<0.001). In addition, decreased expression of miR-149 correlates with lymph node metastasis (*p = 0.046) and TNM stage (**p = 0.0011) (Table 1).

**Table 1:** The relationship between clinical parameters and miR-149 (Mean±SE) expression in primary gastric adenocarcinoma.

Clinical parameters	N(%)	Relative expression	p-value
Age(years)			
≥60	19(43)	0.5326±0.02799	0.37
<60	25(57)	0.4956±0.02878	
Gender			
Male	32(73)	0.5059±0.02507	0.65
Female	12(27)	0.5267±0.03396	
Size			
≥5	26(59)	0.5273±0.02768	0.36
<5	18(41)	0.4889±0.02948	
Histologic differentiation			
Well(W)	13(30)	0.6500±0.02048	
Moderately(M)	16(36)	0.5388±0.01549	P: <0.001***
Poorly (P)	15(34)	0.3627±0.01733	
Lymphatic metastasis			
No	13(30)	0.5738±0.04139	0.046*
Yes	31(70)	0.4855±0.02177	
TNM stage			
Stage I/II	20(45)	0.5810±0.02796	0.0011*
Stage III/IV	24(55)	0.4538±0.02354	

### Ectopic expression of miR-149 inhibits proliferation and induces G0/G1 arrest in AGS and SGC7901 cells

**Figure 2:**
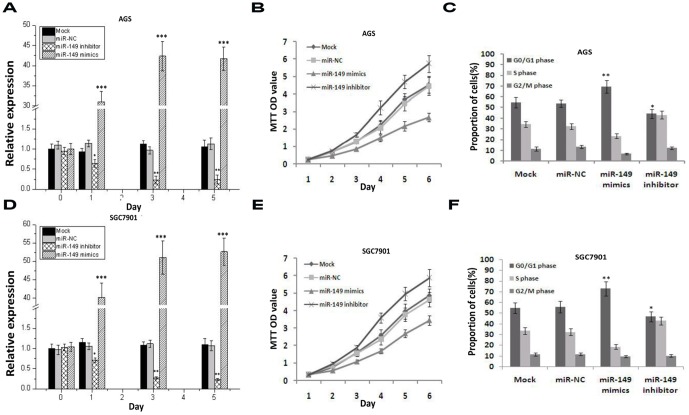
Expression of miR149 inhibits proliferation and induces G0/G1 arrest of GC cell lines. A&D. The level of miR149 was measured by quantitative PCR at designated time (One-way ANOVA analysis, the values indicate the mean ± SEM, fig. 2A, F = 8.429, p = 0.003; fig. 2D, F = 8.595, p = 0.003). B&E. GC cells transfected with miR-149 mimics and inhibitor subjected to MTT assay daily for 6 days (Two-way ANOVA analysis, fig. 2B, F = 27.47, p<0.001; Fig. 2E, F = 44.061, p<0.001). C&F. GC cells cells transfected with miR-149 mimics and inhibitor were collected for FACS analysis after 72 h (The values indicate the mean ± SEM, n = 3, One-way ANOVA, fig. 2C, F = 134.177, p<0.001; Fig. F, F = 58.792, p = 0.003).

To investigate the role of miR-149 in GC cells, we utilized poorly and moderately differentiated GC cell lines, AGS and SGC7901, respectively. AGS and SGC7901 cells were transiently transfected with eithermature miR-149 mimics, inhibitor, mock transfected, or miR-NC. As shown in figure 2A, 2D, quantitative RT-PCR results show that expression of miR-149 mimics or inhibitors significantly upregulate or downregulate the expression of miR-149, respectively, in AGS and SGC7901 cells from the first to fifth day posttransfection (Fig. 2A, F = 8.429, *p* = 0.003; fig. 2D, F = 8.595, *p* = 0.003) compared to NC and mock controls.

AGS and SGC7901 cells transfected with miR-149 mimics and inhibitors showed significantly lower and higher levels of cell proliferation, respectively, than the NC or mock groups as determined by MTT assay (Fig. 2B, F = 27.47, p<0.001; Fig. 2E, F = 44.061, p<0.001). Transfection of AGS and SGC7901 cells with miR-149 mimics results in significant G1 phase arrest in comparison to NC and mock controls (*p*<0.05). Moreover, treatment with miR-149 inhibitors led to fewer AGS and SGC7901 cells in G1 arrest compared to NC and mock controls (*p*<0.05) (Fig. 2C, F = 134.177, *p*<0.001; Fig. 2F, F = 58.792, *p* = 0.003).

### *ZBTB2* is a target of miR-149

**Figure 3:**
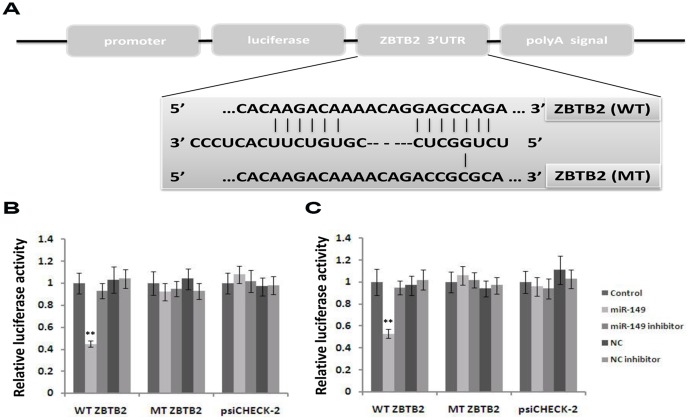
Validating the predicted binding sites between miR149 and ZBTB2. A. The schematic diagram shows the construct of Luc-ZBTB2 3′UTR and Luc-ZBTB2 3′Mut UTR.Both Luc-ZBTB2 3′UTR and Luc-ZBTB2 3′Mut UTR were cloned into a pmirGLO plasmid downstream of the firefly luciferase coding region between the PmeI and XbaI sites. B&C. AGS cells (B) or SGC7901 cells(C) were co-transfected with the psiCHECK-2 constructs containing either ZBTB2 3′UTR or ZBTB2 3′Mut UTR and either the miR-149 inhibitor or the miR-149 mimics for 48 h. Values indicate the relative luciferase activity after normalization to Renilla luciferase activity(The values indicate the mean ± SEM, n = 3, One-way ANOVA analysis,**p<0.01).

Previous data suggest that miR-149 might be a suppressor of GC cell growth by targeting genes that control proliferation and cell cycle progression (33–34)(38). Thus, we searched for additional potential targets of miR-149 from TargetScanHuman database. We identified numerous potential miR-149 target genes, including *ZBTB2*, a POK family transcription factor and potent repressor of the ARF-HDM2-p53-p21 pathway(41). *ZBTB2* was found to have putative miR-149 binding sites within its 3′UTR (Fig. 3A). Luciferase reporter assays were performed to verify whether *ZBTB2* is a direct target of miR-149 using AGS and SGC7901 cells. We co-transfected AGS and SGC7901 cells respectively with a psiCHECK-2 vector containing either 3′UTR for ZBTB2 or mutated 3′UTR for ZBTB2, and mimics of miR-149 or inhibitors of miR-149. Wild-type and mutant ZBTB2-3′UTR containing the putative binding site of miR-149 were cloned into psiCHECK-2 vector downstream from luciferase gene (Fig. S1). Introduction of miR-149 significantly reduced the luciferase activity from the ZBTB2 3′UTR reporter vector (Fig. 3B–3C, *p*<0.001), but did not affect the luciferase activity from the mutant ZBTB2-3′UTR reporter vector. These data suggest that miR-149 directly regulates *ZBTB2* expression levels. Furthermore, there is no significant decrease in relative luciferase activity in cells co-transfected with miR-149 inhibitor or 3′UTR-ZBTB2/psiCHECK-2 vector (Fig. 3B–3C). It is maybe because of the lack of interaction between 3′UTR of ZBTB2 and endogenous miR-149, hence, decreased miR-149 expression did not increase luciferase activity from the ZBTB2-3′UTR reporter vector. These results further confirm that miR-149 suppresses ZBTB2 expression by targeting the 3′-UTR of *ZBTB2* mRNA.

### miR-149 and ZBTB2 expression is negatively correlated in GC cells and clinical samples

**Figure 4:**
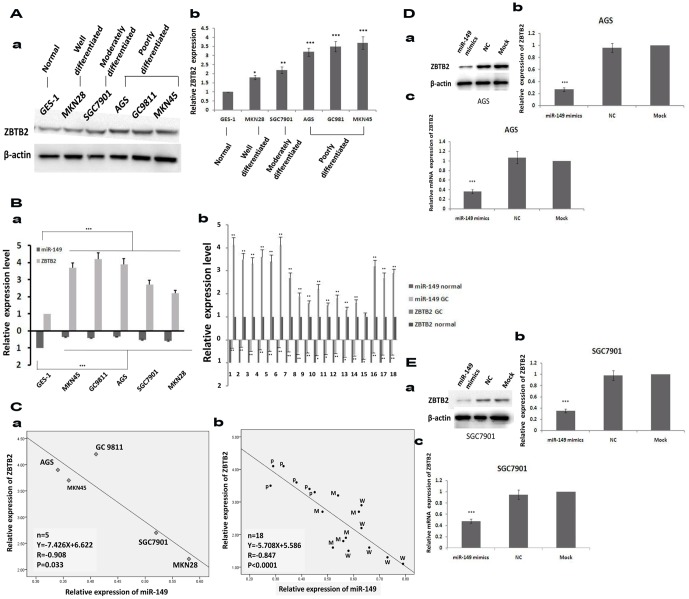
Expression of miR-149 and ZBTB2 negatively correlate in GC cell lines and clinical samples. A. Expression of ZBTB2 in GC cells and the normal gastric epithelial cell were examined by western blotting (a) and shown as mean ± SEM(b, normalized to actin, One-Way ANOVA analysis, F = 167.843, *** p<0.001). B. Expression of ZBTB2 and expression of miR-149 in gastric cells (a, the values indicate the mean ± SEM, One-Way ANOVA analysis, F = 283.355, p<0.001) and clinical samples (b) analyzed by quantitative PCR(t-test, the values indicate the mean ± SEM,*, p<0.05;** p<0.01;*** p<0.001). C. Scatter plots showing the negative linear correlation between the mRNA expression of ZBTB2 and that of miR-149 in gastric cells: MKN45, GC9811, AGS, SGC7901 and MKN28 (a) and in 18 gastric clinical samples (b) (P: poorly differentiated; M: moderately differentiated; W: well differentiated). D. Expression of ZBTB2 examined by western blotting (a, b: the values indicate the mean ± SEM, normalized to actin, One-Way ANOVA analysis, F = 682.839, *** p<0.001) and quantitative PCR(c, the values indicate the mean ± SEM, F = 1420.125, *** p<0.001) after being treated with miR-149 mimics in AGS cells. E. Expression of ZBTB2 examined by western blotting (a, b: the values indicate the mean ± SEM, normalized to actin, One-Way ANOVA analysis, F = 2459.400, *** p<0.001) and quantitative PCR(c, the values indicate the mean ± SEM, One-Way ANOVA analysis, F = 925.875, *** p<0.001) after being treated with miR-149 mimics in SGC7901 cells.

To further investigate the relationship between miR-149 and ZBTB2, we examined the expression of ZBTB2 in GC cells using western blotting and in GC tissues using immunohistochemistry. It was found that GC cells have significantly higher expression level of ZBTB2 than the normal gastric epithelial cell, GES-1 (Fig. 4A a–b F = 167.843, *p*<0.001). ZBTB2 expression negatively correlated with the differentiation degree of the GC cells; poorly differentiated GC tissues showed a significant higher ZBTB2 expression level than well differentiated GC tissues and matched normal gastric tissues (Fig. S2A–B, F = 117.280, *p*<0.001). *ZBTB2* mRNA expression levels are coincident with the protein levels (Fig. 4B a, F = 283.355, *p*<0.001). Furthermore, the expression of miR-149 and ZBTB2 is inversely correlated (Fig. 4B b, *p* = 0.033, R = −0.908). We then measured the expression levels of miR-149 and *ZBTB2* using quantitative RT-PCR in 5 GC cell lines (MKN45, GC9811, AGS, SGC7901, and MKN28) (Fig. 4C a) and 18 GC clinical samples (6 poorly, 6 moderately, and 6 well differentiated samples) (Fig. 4C b). Results confirm that the mRNA level of *ZBTB2* negatively correlates with miR-149 expression (Fig. 4C a, *p* = 0.033, R = −0.847; Fig. 4C b, *p*<0.001, R = −0.908). Moreover, AGS (Fig. 4D) and SGC7901 cells (Fig. 4E) transfected with miR-149 mimics have significantly decreased expression of ZBTB2 at the protein levels (panels a–b) and mRNA levels (panel c) compared to mock or NC cells (*p*<0.001). Our findings indicate that miR-149 may directly regulate the expression of *ZBTB2*.

### miR-149 suppresses GC cell proliferation and cell cycle progression by targeting *ZBTB2*

**Figure 5:**
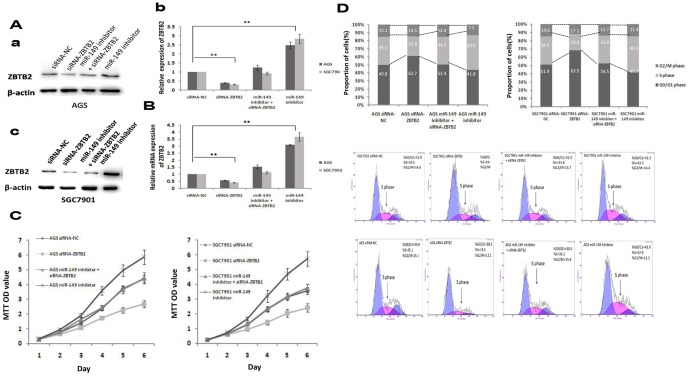
ZBTB2 mediates the function of miR-149 by suppressing GC cell proliferation and cell cycle progression. A. Expression of ZBTB2 examined by western blotting (a–c, normalized to actin, the values indicate the mean ± SEM, One-Way ANOVA analysis, for AGS, F = 3163.690, *p*<0.001; for SGC7901, F = 3979.861, *p*<0.001). B. Expression of ZBTB2 examined by quantitative PCR (the values indicate the mean ± SEM, One-Way ANOVA analysis, for AGS, F = 3785.817, *p*<0.001; for SGC7901,F = 7392.941, *p*<0.001). C. GC cells treated with siRNA-NC, siRNA-ZBTB2, miR-149 inhibitor+siRNA-ZBTB2,or miR-149 inhibitor subjected to MTT assay daily for 6 days (the values indicate the mean ± SEM, Two-way ANOVA analysis, for AGS,F = 18.553, *p*<0.001; for SGC7901,F = 24.857, *p*<0.001). D. GC cells transfected cells treated with siRNA-NC, siRNA-ZBTB2, miR-149 inhibitor+siRNA-ZBTB2,or miR-149 inhibitor were collected for FACS analysis after 72 h(the values indicate the mean ± SEM, n = 3, *, *p*<0.05;** *p*<0.01;*** *p*<0.001).

Next we confirmed that miR-149-mediated proliferation inhibition and induction of cell cycle arrest in GC cells requires targeting of *ZBTB2*. *ZBTB2* expression was knocked-down using siRNAs in AGS and SGC7901 cells. Western blotting (Fig. 5A, a–c) and quantitative PCR analysis showed that depletion of *ZBTB2* by siRNA transfection could be compensated significantly by miR-149 inhibitor and a marked increase of ZBTB2 was seen in cells transfected with miR-149 inhibitors(Fig. 5B, *p*<0.001). MTT assay shows that proliferation of cells transfected with siRNA-ZBTB2 was suppressed and the proliferation of cells transfected with miR-149 inhibitors increased significantly compared to cells transfected with siRNA-NC (control) and siRNA-ZBTB2+miR-149 (Fig. 5C). Furthermore, inhibition of *ZBTB2* expression promotes G0/G1 arrest and suppresses the G0/G1 cell cycle arrest induced by miR-149 inhibitor treatment in AGS and SGC7901 cells compared to NC control cells (Fig. 5D–5E, *p*<0.001). Our findings suggest that miR-149 inhibits GC cell proliferation and cell cycle progression by inhibiting *ZBTB2* expression.

### Introduction of miR-149 altered the expression of ZBTB2 and cell-cycle-related proteins

**Figure 6:**
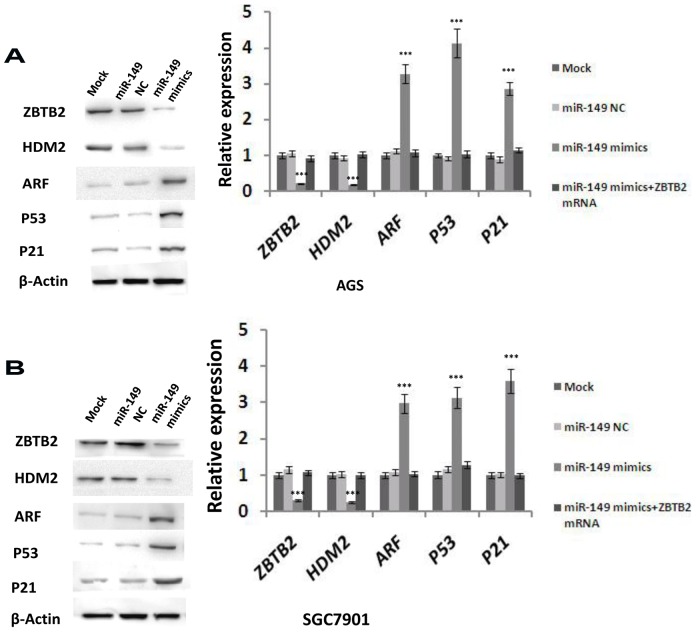
Expression of miR-149 alters the expression of ZBTB2 and cell-cycle-related proteins. A, Expression of ZBTB2(F = 629.069,*p*<0.001), HDM2(F = 869.909,*p*<0.001), ARF(F = 1425.913, *p*<0.001), p53(F = 6608.889, *p*<0.001) and p21(F = 1818.737, *p*<0.001) in SGC7901 cell were examined by western blotting (a) and shown as mean ± SEM(b, normalized to actin, One-way ANOVA analysis, n = 3,*** *p*<0.001). B, Expression of ZBTB2(F = 527.382, *p*<0.001), HDM2(F = 631.259, *p*<0.001), ARF(F = 1517.081, *p*<0.001), p53(F = 1753.000, *p*<0.001) and p21(F = 2751.400, *p*<0.001) in AGS cell were examined by western blotting (a) and shown as mean ± SEM(b, normalized to actin, One-way ANOVA analysis, n = 3,*** *p*<0.001).

ZBTB2 has been reported to be a potent repressor of the ARF-HDM2-p53-p21 pathway, which is important in cell cycle regulation (41). To investigate the regulation of these proteins by miR-149 in GC cells, we transfected AGS and SGC7901 cells with miR-149 mimics or miR-NC and performed RT-PCR and western blotting analysis for ZBTB2 target genes. As shown in Figure 6A–6B, ectopic expression of miR-149 downregulates the expression of ZBTB and HDM2, and upregulates the expression of ARF, p53, and p21 (*p*<0.001). These data are consistent with the function of ZBTB2 in repressing the transcription of ARF, p53, and p21 and activating the expression of HDM2(41). Moreover, ectopic expression of ZBTB2 by transfection reversed the miR-149-mediated reduction in HDM2 expression and increase in ARF, p53, and p21 expression in AGS and SGC7901 cells (Fig. 6A–B). Ectopic ZBTB2 expression was confirmed in transfected AGS and SGC7901 cells (Fig. S3, SGC7901 cells, F = 806.365, *p*<0.001; AGS cells, F = 1436.300, *p*<0.001). ARF, p53 and p21 promote cell cycle progression therefore, their downregulation by expression of miR-149 mimics explains the G0/G1 phase arrest in AGS and SGC7901 cells.

## Discussion

Increasing evidence suggests that miRNAs play a critical role in carcinogenesis and cancer progression [21]. Altered miRNA expression levels have been implicated in the initiation and development of tumors; modulation of miRNAs that function as negative regulators of oncogenes or tumor suppressors results in promotion of cancer cell proliferation and growth [28]–[32]. Previous studies have shown that the role of miR-149 in the progression of various types of tumors is controversial [33]–[36], [38]. The expression pattern and targets of miR-149 vary in different types of tumors. miR-149 expression is downregulated in some tumors, functioning as a tumor suppressor by targeting oncogenes in some cancers. For example, in clear cell renal cell carcinoma miR-149 is downregulated and its likely targets were identified as KCNMA1 and LOX(35). Furthermore, miR-149 is also downregulated in glioblastoma cells and has been proposed to act as a tumor suppressor by targeting RAP1B, CD47, CCN1, and NXF1 [36]. In contrast, miR-149 can also function as a tumor suppressor by targeting certain oncogenes. Detection of miRNA expression profiles in different clinical stages of nasopharyngeal carcinoma (NPC) and NPC lymph node metastasis shows that the expression of miR-149 is upregulated in NPC and expression of its target gene, *Smad2*, is decreased with the progression of NPC [38]. A similar oncogenic role for miR-149 was also seen in melanoma in which upregulation of miR-149 causes downregulation of GSK3-α and upregulation of Mcl-1, resulting in apoptotic resistance [34]. Thus far, little is known about the role of miR-149 in gastric cancer. Here, we report that miR-149 expression is remarkably reduced in multiple gastric cancer cell lines and clinical specimens compared with the normal gastric epithelial cell and matched adjacent normal tissues, respectively. Importantly, the level of miR-149 expression is associated with the differentiation degree of GC cells and specimens; poorly differentiated GC cells or specimens have lower miR-149 expression compared to well differentiated samples. Ectopic expression of miR-149 inhibits cells proliferation and cell cycle progression by targeting *ZBTB2* in AGS and SGC7901 cells. miR-149 and ZBTB2 expression are negatively correlated in GC cells and clinical specimens, and overexpression of miR-149 causes downregulation of *ZBTB2*. Depletion of ZBTB2 results in inhibition of AGS and SGC7901 cells proliferation and cell cycle progression. Collectively, these results demonstrate, for the first time, that ZBTB2 may function as an oncogene in the tumorigenesis of gastric cancer.

Our data show that introduction of miR-149 into GC cells induces cell cycle arrest, suggesting that miR-149 targets may be implicated in cell cycle regulation. ZBTB2 is a POK family transcription factor which can suppress the ARF-HDM2-p53-p21 pathway [41]. ARF, a tumor suppressor [42], can be induced by sustained mitogenic stimulation and plays a key role in activating p53 independently and controlling the stability of p53 through interaction with HDM2 [43], an important regulator of p53. The tumor suppressor p53 plays a critical role in the maintenance of cell homeostasis through inducing cell cycle arrest and apoptosis when cells are subjected to stressors, such as hypoxia, DNA damage, and telomere dysfunction [44]. p21, a p53 target gene, mediates cell cycle G1 phase arrest through binding and inhibiting the activity of cyclin-CDK2 or CDK1 complexes [45]. ZBTB2 can repress the transcription of ARF, p53, and p21, and induce HDM2 expression [41]. To validate whether the cell cycle effects induced by miR-149 expression is mediated by ZBTB2 loss, we examined expression of HDM2, ARF, p53 and p21 of AGS and SGC7901 cells transfected with miR-149 mimics. We found that ectopic expression of miR-149 results in increased expression of ARF, p53, and p21 and decreased expression of HDM2 and ZBTB2. These results support the idea that miR-149 exerts its role of inhibiting GC cells proliferation and cell cycle progression by targeting *ZBTB2* thereby modulating the expression of downstream regulators of cell cycle progression.

In summary, our data indicate that miR-149 may function as a tumor suppressor in gastric cancer cells and plays an important role in inhibiting *ZBTB2*. Therefore, downregulation of miR-149 promotes GC cell proliferation and cell cycle progression. Moreover, our results show that ZBTB2 may function as an oncogene in the development of gastric cancer. However, given the fact that each miRNA may regulate many target genes which can affect carcinogenesis in different ways, more studies are needed to investigate other miR-149 targets which may have critical roles in GC tumorigenesis. The present study also provides novel insight into the role of miR-149 in human gastric cancer progression and indicates that miR-149 may serve as a therapeutic target for gastric cancer treatment.

## Materials and Methods

### Ethics Statement

For tissue samples, written informed consent was obtained from patients. The procedures used in this study were approved by the Institutional Review Board of the Fourth Military Medical University and was conformed to the Helsinki Declaration, and to local legislation.

### Cell lines and culture conditions

Gastric cancer lines AGS, MKN28, MKN45 were bought from AGCC and, GC9811 and SGC7901 cell lines were obtained from Beijing Institute of Oncology. All the cell lines were maintained in our institute according to recommended protocols. Cells were cultured in RPMI-1640 medium (Invitrogen, Carlsbad, CA, USA) supplemented with 10% fetal bovine serum (FBS) (Invitrogen, Carlsbad, CA, USA)at 37°C in a 5% CO_2_ incubator.

### Human specimens

All experimental procedures were approved by the Institutional Review Board of the Fourth Military Medical University. Written informed consent was obtained for all patient samples.Human gastric cancer specimens (n = 44) and matched adjacent non-tumor specimens were obtained from patients at Xijing Hospital of Digestive Diseases, the Fourth Military Medical University, with informed consent from each patient.

### RNA purification, cDNA synthesis, and quantitative real-time PCR (qRT-PCR)

Total RNA of cultured cells was extracted with TRIzol reagent (Invitrogen, Carlsbad, CA, USA) according to the manufacturer's protocol and RNAs were stored at −80°C before qRT-PCR analysis. Mature miR-149 expression was detected using a mirVana TM qRT-PCR miRNA Detection Kit (Ambion Inc. Austin, Texas), with U6 as an internal control. ZBTB2 expression was detected with primers F: 5′ACTCGGGCGAGATCCACGGC 3′, R: 5′ ACGCAGGCTGCTCATCGGAG 3′, and GAPDH was used as an internal control. PCR products were separated on an ethidium bromide-stained 1.5% agarose gel and visualized with UV.

### Cell transfection

The human miR-149 duplex mimic and inhibitor (miR-149) and negative control oligonucleotide duplex mimic (miR-NC) were designed and provided by Ribobio (Guangzhou, Guangdong, China). The small interfering RNA (siRNA) for ZBTB2 and the negative control RNA (siRNA-NC) were synthesized and purified by Genepharma(Shanghai,China). miRNAs, siRNAs and ZBTB2 cDNA plasmid (FulenGen Co. China)were transfected by LipofectamineTM 2000 reagent (Invitrogen, Carlsbad, CA, USA) according to the manufacturer's protocol. The ZBTB2 siRNA sequence: F: 5′ GCUGGCUUCUUUCCAAGUU dTdT 3′, R: 5′ AACUUGGAAAGAAGCCAGC 3′; The NC siRNA sequence: F: 5′-UUCUCCGAACGUGUCACGUTT -3′,R: 5′-ACGUGACACGUUCGGAGAATT -3′.

### miRNA target prediction

To find potential miRNA target genes, TargetScanHuman website (http://www.targetscan.org/) was used, the binding free energy was calculated and biding sites were analyzed using http://bibiserv.techfak.uni-bielefeld.de/rnahybrid website.

### Vector constructs and luciferase reporter assay

To construct ZBTB2-3′UTR plasmid, a wild-type 3′-UTR fragment of human ZBTB2 mRNA (1238–1244 nt, Genbank accession no. NM_020861.1) containing the putative miR-7 binding sequence was amplified by RT-PCR and cloned into the site between Xho I and Not I downstream of the luciferase reporter gene of the psiCHECK™ vector (Promega, USA). A mutant of the single miR-7 binding site (5′- GAGCCAG-3′ to 5′- ACCGCGC-3′) in the 3′-UTR of ZBTB2 was included by Site-Directed Mutagenesis Kit (SBS Genetech, Beijing, China). Wild and mutant types of pmirGLO-ZBTB2-UTR vectors were validated by DNA sequencing.

The nucleotide sequences of primers for ZBTB2-3′UTR (MT) clone: mutZBTB2F:5′GGCCTCACAAGACAAAACAGACCGCGCAAGTAAGGACTGAAGGAGAA 3′ mutZBTB2R:5′TTCTCCTTCAGTCCTTACTTGCGCGGTCTGTTTTGTCTTGTGAGGCC 3′

The nucleotide sequences of primers for ZBTB2-3′UTR (WT) clone: ZBTB2XhoIF:5′CCGCTCGAG ATGTCACTTATCTTTTTAAAAAACTCTCA 3′ ZBTB2NotIR:5′ATAAGAATGCGGCCGC TACTCATTTAATTAAACGTTTATTC 3′ ZBTB2XhoIF2:5′CCGCTCGAGATGTCACTTATCTTTTTAAAAAACTCTCATTTTTACAAAGACTATC 3′ ZBTB2F3:5′CCGCTCGAG CTGTAGTTCTCCATGGCATGATAC 3′.

Cells were transfected with the miR-149 mimics, NC and pmirGLO plasmid in 24-well plates using lipofectamine™ 2000 (Invitrogen) according to the instructions. 48 h later, cells were harvested and analyzed for luciferase activity using the Dual-Luciferase Reporter Assay System (Promega, USA) and detected by the GloMaxTM 20/20 detection system (E5331, Promega).

### Western blotting

Total protein from cultured cells were lysed by Lysis Buffer containing PMSF on ice. Then protein were electrophoresed through 12% SDS polyacrylamide gels and were then transferred to a PVDF membrane (Millipore). Membranes were blocked with 5% non-fat milk powder at room temperature for 1 h and incubated overnight with primary antibodies. Membranes were incubated with secondary antibodies labeled with HRP for 1 h at room temperature after three 10 min washes in TBS-T(triethanolaminebuffered saline solution with Tween). Finally, the signals were detected using ECL kit (Pierce Biotech., Rockford, IL, USA) and the membranes were scanned and analyzed using a Bio-Rad ChemiDoc XRS+ imaging system with imaging software (version quantity 1). The protein expression was normalized to an endogenous reference (Actin) and relative to the control. The Spectra multicolor broad-range protein ladder (Fermentas) was used as molecular marker. All the antibodies used in western blot assay are listed in table S1.

### Immunohistochemistry and Immunohistochemical scoring

Paraffin sections, 4-µm in thickness, were baked for 2 h at 65°C and deparaffinized. Antigen retrieval was performed using citrate sodium buffer (PH 7.2) at 95°C for 15 minutes and then slides were cooled at room temperature for 30 minutes. After being treated with 3% hydrogen peroxide for 15 minutes to block the endogenous peroxidase, the sections were treated with normal goat serum confining liquid for 30 minutes to reduce non-specific binding and then rabbit polyclonal anti-ZBTB2 (1∶500, Bioss Co. China) was incubated the sections for 12 h at 4°C.After rewarming for 1 h and washing for 5 times, sections were incubated with secondary antibody for 30 minutes at room temperature. Diaminobenzidine(DAB) was used for color reactions. Subsequent immunohistochemical staining was scored as previously described [46].

### MTT assay

Cells were transfected with 100 nM miR-149 inhibitor (Genepharma,Shanghai,China),mimics(Ribobio,Guangzhou,Guangdong,China) or 100 nM siRNA-ZBTB2 (Genepharma).Twenty-four later, cells were seeded in 96-well plates(2×103/well). The viability of cells was examined by MTT (3-2, 5-diphenyl tetrazolium bromide) assay (Sigma,USA) daily for 6 days.

### Cell cycle assay by flow cytometry

Gastric cancer cell lines, AGS and SGC7901 were grown in RPMI-1640 medium (Invitrogen, Carlsbad, CA, USA) supplemented with 10% FBS in 6-well plates. Transfection were performed when cells reached 80%%–90% confluency. Subsequently, cells were harvested, washed twice using PBS, and fixed in 70% ethanol at 4°C overnight. Then cells were incubated with propidium iodide at room temperature for 1 h and were analyzed by flow cytometry using a FACScan flow cytometer (BD Biosciences, Mountain View, CA). These data were analyzed by using Flowjo (Flowjo).

### Statistical analysis

Data were expressed as Mean±SEM of three independent experiments. For statistical tests, SPSS statistical software package, version17.0 (SPSS, Chicago, IL, USA) was used. The student's t-test, the one-way ANOVA and two-way ANOVA test were performed for MTT assays, quantitative PCR and tumor growth curve. The correlation between miR-149 and ZBTB2 was analyzed with Spearman rank correlation. P values <0.05 were considered statistically significant.

## Supporting Information

Figure S1 **Wild-type and mutant** ***ZBTB2*** **-3′UTR containing the putative binding site of miR-149 were cloned into psiCHECK-2 vector.** A. *ZBTB2*-3′UTR was amplified from genomic DNA of AGS. B. Lane 1,3, 5: Recombinant plasmids of *ZBTB2-1, ZBTB2-2, ZBTB2-3* respectively; lane 2,4,6: Results of enzyme digestion of recombinant plasmids of *ZBTB2-1, ZBTB2-2, ZBTB2-3* respectively. Results showed that *ZBTB2*-1/2/3 have been successfully inserted into the vectors.(M1: DL2000 DNA Marker; M2: DL1 kb DNA Marker; ZTBT2-1/2/3 bands: 1406 bp; Vectors bands: 6.1 Kb). C. M1: 1 kb DNA Ladder Marker. Lane 1: amplification of mut*ZBTB2*F1/R1 (negative control without Taq enzyme); Lane 2: amplification of mut*ZBTB2*F1/R1. One band of mut*ZBTB2* (7.6 Kb) demonstrated the successful PCR of mutant amplification. D. Sequencing results of WT-*ZBTB2* and MT –*ZBTB2*.
(TIF)

Figure S2 **ZBTB2 expression in human gastric cancer specimens and matched adjacent normal tissues.** A. Representative images shown are positive immunohistochemical staining of ZBTB2 in human gastric cancer specimens (b–c) and matched adjacent normal tissues (a)(magnification 200×). B. Immunohistochemical staining was scored as previously described [46]; statistical analysis of data on the ZBTB2 expression difference between human gastric cancer specimens and matched adjacent non-cancerous tissues (One-Way ANOVA analysis, F = 117.280, *** *p*<0.001).
(TIF)

Figure S3 **Ectopic** ***ZBTB2*** **mRNA expression increases ZBTB2 protein expression in GC cell lines.** A. Ectopic ZBTB2 mRNA expression increases ZBTB2 protein expression in SGC7901 cell line.(The values indicate the mean±SEM, n = 3, One-Way ANOVA analysis, F = 806.365,*** *p*<0.001). B. Ectopic ZBTB2 mRNA expression increases ZBTB2 protein expression in SGC7901 cell line. (The values indicate the mean±SEM, n = 3, One-Way ANOVA analysis, F = 1436.300, *** *p*<0.001).
(TIF)

Table S1 **Antibodies used for the experiments.**
(DOC)
