# Supplementary figures and images for: MicroRNA-149 Inhibits Proliferation and Cell Cycle Progression through the Targeting of ZBTB2 in Human Gastric Cancer
Source: PLoS One. 2012 Oct 29;7(10):e41693. doi: 10.1371/journal.pone.0041693 (PMC3483266; doi:10.1371/journal.pone.0041693)

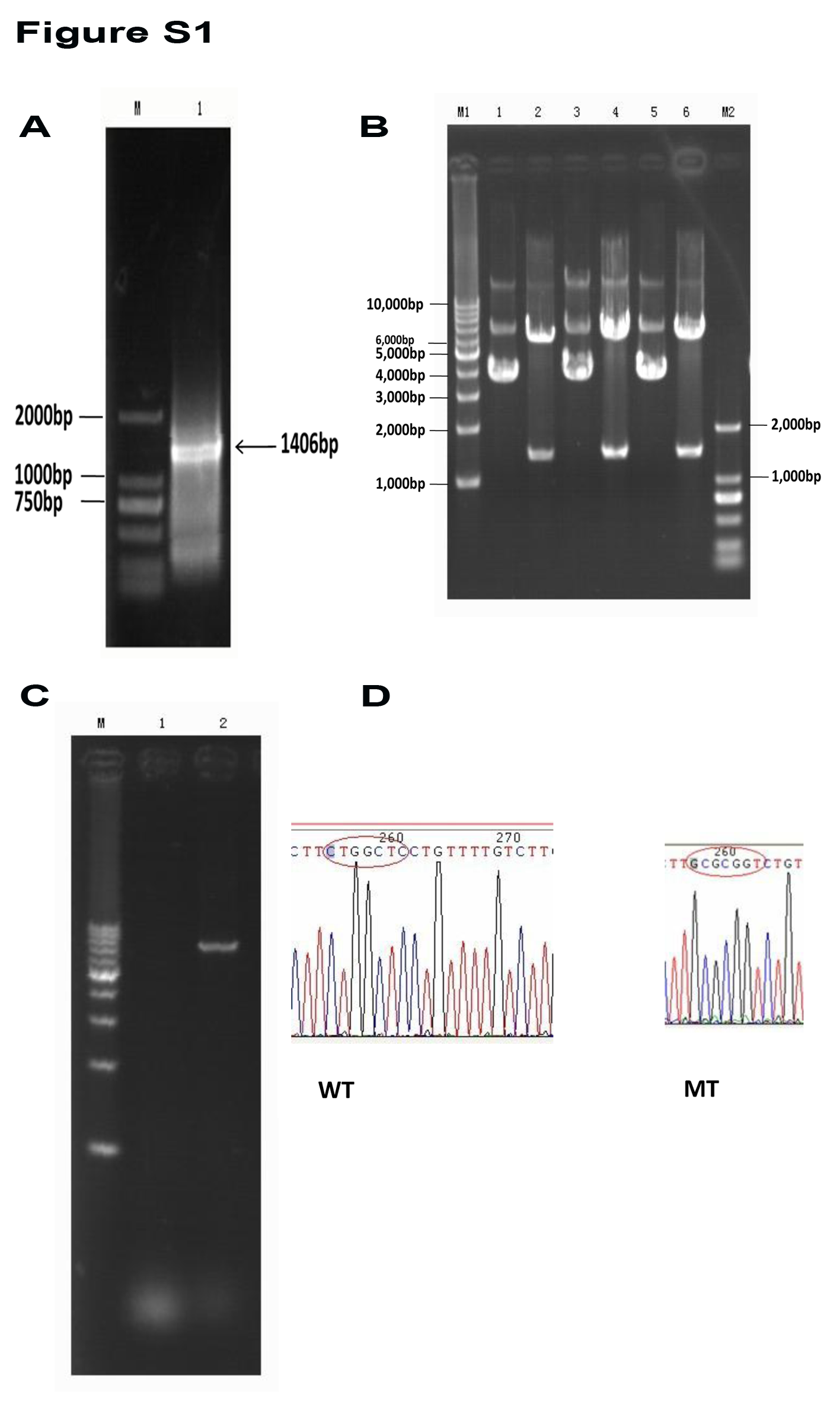

Supplement: Figure S1 — Wild-type and mutant ZBTB2 -3′UTR containing the putative binding site of miR-149 were cloned into psiCHECK-2 vector. A. ZBTB2-3′UTR was amplified from genomic DNA of AGS. B. Lane 1,3, 5: Recombinant plasmids of ZBTB2-1, ZBTB2-2, ZBTB2-3 respectively; lane 2,4,6: Results of enzyme digestion of recombinant plasmids of ZBTB2-1, ZBTB2-2, ZBTB2-3 respectively. Results showed that ZBTB2-1/2/3 have been successfully inserted into the vectors.(M1: DL2000 DNA Marker; M2: DL1 kb DNA Marker; ZTBT2-1/2/3 bands: 1406 bp; Vectors bands: 6.1 Kb). C. M1: 1 kb DNA Ladder Marker. Lane 1: amplification of mutZBTB2F1/R1 (negative control without Taq enzyme); Lane 2: amplification of mutZBTB2F1/R1. One band of mutZBTB2 (7.6 Kb) demonstrated the successful PCR of mutant amplification. D. Sequencing results of WT-ZBTB2 and MT –ZBTB2. (TIF) [file pone.0041693.s001.tif]

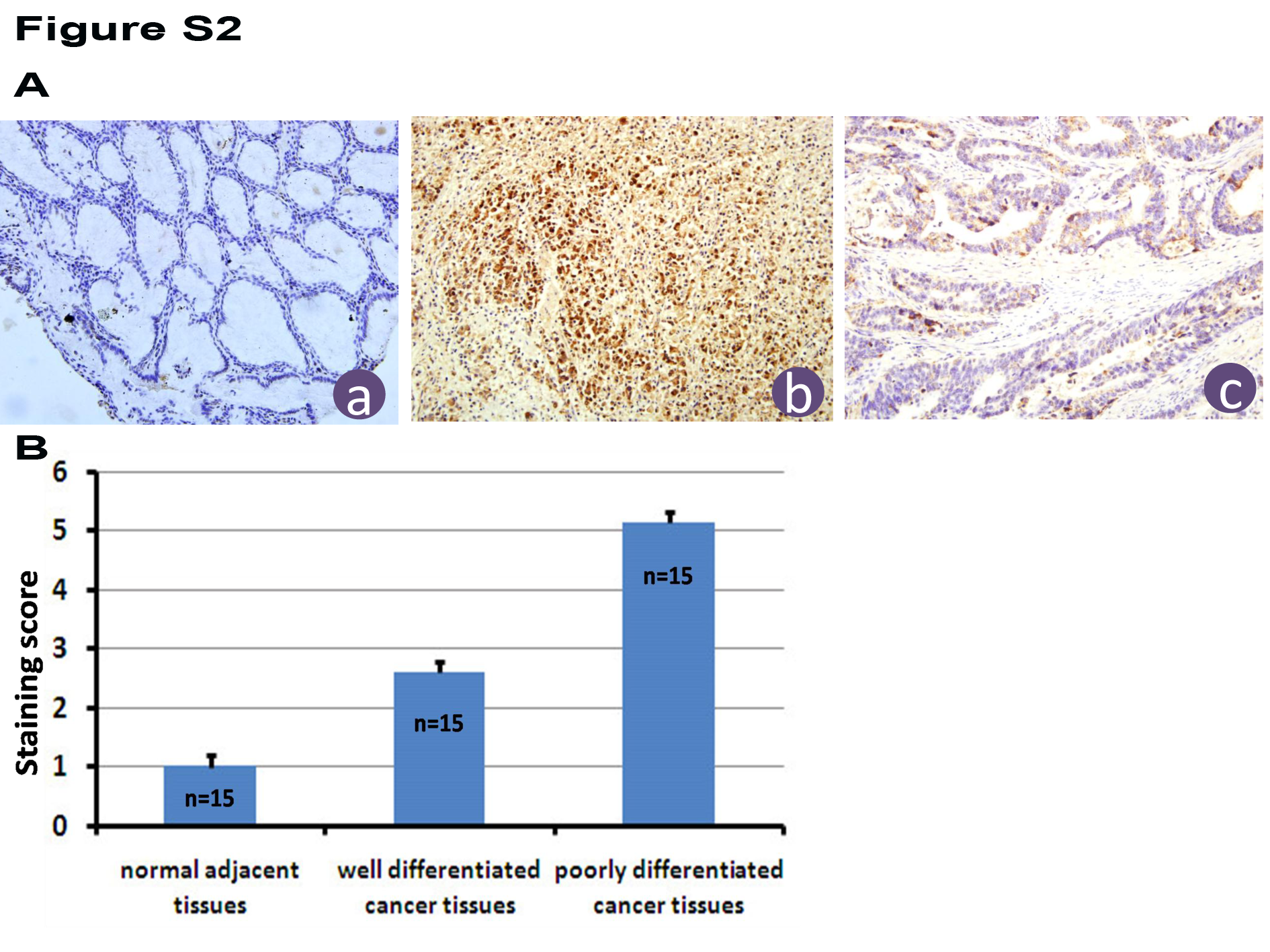

Supplement: Figure S2 — ZBTB2 expression in human gastric cancer specimens and matched adjacent normal tissues. A. Representative images shown are positive immunohistochemical staining of ZBTB2 in human gastric cancer specimens (b–c) and matched adjacent normal tissues (a)(magnification 200×). B. Immunohistochemical staining was scored as previously described [46]; statistical analysis of data on the ZBTB2 expression difference between human gastric cancer specimens and matched adjacent non-cancerous tissues (One-Way ANOVA analysis, F = 117.280, *** p<0.001). (TIF) [file pone.0041693.s002.tif]

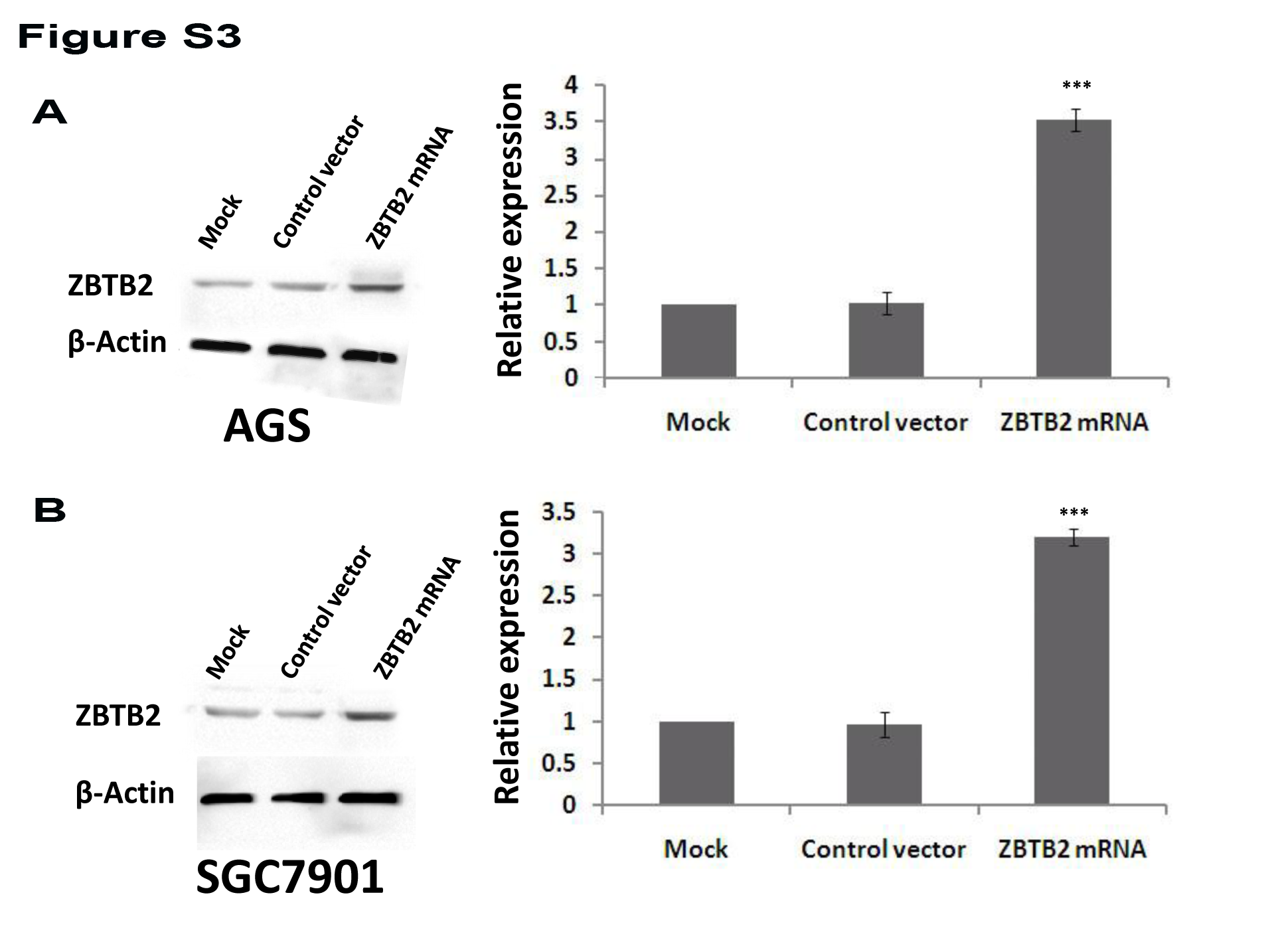

Supplement: Figure S3 — Ectopic ZBTB2 mRNA expression increases ZBTB2 protein expression in GC cell lines. A. Ectopic ZBTB2 mRNA expression increases ZBTB2 protein expression in SGC7901 cell line.(The values indicate the mean±SEM, n = 3, One-Way ANOVA analysis, F = 806.365,*** p<0.001). B. Ectopic ZBTB2 mRNA expression increases ZBTB2 protein expression in SGC7901 cell line. (The values indicate the mean±SEM, n = 3, One-Way ANOVA analysis, F = 1436.300, *** p<0.001). (TIF) [file pone.0041693.s003.tif]
