# Supplementary material for: MicroRNA-149 Inhibits Proliferation and Cell Cycle Progression through the Targeting of ZBTB2 in Human Gastric Cancer
Source: PLoS One. 2012 Oct 29;7(10):e41693. doi: 10.1371/journal.pone.0041693 (PMC3483266; doi:10.1371/journal.pone.0041693)
Supplement: Table S1 — Antibodies used for the experiments. (DOC) [file pone.0041693.s004.doc]

| **Supplementary Table 1 :Antibodies used for the experiments** | | | | |
| --- | --- | --- | --- | --- |
| **Primary antibody** | **Species raised in** | **Supplier** | **Dilution for WB** | **Band Size** |
| ZBTB2 | Rabbit | Abcam(ab83855) | 1:100 | 50 kDa |
| HDM2 | Mouse | Santa Cruz(sc-56154) | 1:500 | 90 kDa |
| ARF | Rabbit | Santa Cruz(sc-53167) | 1:200 | 21 kDa |
| p53 | Rabbit | Abcam(ab2433) | 1:200 | 55 kDa |
| P21 | Rabbit | Abcam(ab7960) | 1:1000 | 18 kDa |
| Actin | Mouse | Santa Cruz (sc-8432) | 1:200 | 43 kDa |
| anti-mouse IgG | Goat | Cell signaling#7076 | 1:5000 | \ |
| anti-rabbit IgG | Sheep | Sigma(F7512) | 1:5000 | \ |
